# Association of Vitamin D Deficiency as an Independent Risk Factor for Myocardial Infarction and Its Therapeutic Implications: A Systematic Review

**DOI:** 10.7759/cureus.77375

**Published:** 2025-01-13

**Authors:** Abirami Balasubramanian, Keerthi Kunchala, Aaisha Shahbaz, Akankshya Kar, Jawahar Sankar, Sunethra Anand, Mary Attalla, Mariam Hassan, Pareesa K Mehmood, Anusha Kunapuli, Sai Theja Voruganti, Humza F Siddiqui

**Affiliations:** 1 Internal Medicine, Stanley Medical College, Chennai, IND; 2 Internal Medicine, Sri Venkateswara Medical College, Tirupati, IND; 3 Trauma and Orthopedic Surgery, University Hospitals Birmingham NHS Foundation Trust, Birmingham, GBR; 4 Medicine, SRM Medical College Hospital and Research Centre, Chennai, IND; 5 Medicine, Chengalpattu Medical College and Hospital, Chennai, IND; 6 Internal Medicine, Chengalpattu Medical College and Hospital, Chennai, IND; 7 Medicine, Saba University School of Medicine, Saba, NLD; 8 Medicine, Saba University School of Medicine, Overland Park, USA; 9 Medicine, Dow International Medical College, Dow University of Health Sciences, Karachi, PAK; 10 Internal Medicine, People’s College of Medical Sciences and Research Centre, Bhopal, IND; 11 Medicine, Kamineni Institute of Medical Sciences, Hyderabad, IND; 12 Internal Medicine, Jinnah Postgraduate Medical Centre, Karachi, PAK

**Keywords:** cardiovascular diseases, hypovitaminosis d, myocardial infarction, non-st segment elevation myocardial infarction (nstemi), st-elevation myocardial infarction (stemi), vitamin d deficiency, vitamin d receptor, vitamin d supplementation

## Abstract

Vitamin D deficiency is a highly prevalent condition globally and has emerged as a significant risk factor for the development of coronary artery disease (CAD). Although its role in the regulation of phospho-calcic metabolism is well established, its involvement in the evolution of myocardial infarction (MI) is yet to be fully understood. Vitamin D deficiency is a manageable condition, and a reasonable time to assess and commence treatment is crucial, as the patient presents with acute myocardial infarction (AMI). This article discusses the various mechanisms through which vitamin D may prevent MI, including its role in the development of atherosclerosis, as well as its cardioprotective effects. Cardiomyocytes and vascular smooth muscle have exhibited the presence of the vitamin D receptor (VDR) on the surface. Vitamin D deficiency also plays a crucial role in upregulating inflammatory cytokines and activating the renin-angiotensin-aldosterone system (RAAS).

This review aims to explore the implications of vitamin D deficiency on MI by assessing multiple observational studies and randomized controlled trials. The findings suggest a positive correlation between low serum vitamin D levels and the incidence of MI, based on observational studies. Additionally, studies reflect that hypovitaminosis D predisposes AMI patients to post-infarction complications and cardiac remodeling. Contrastingly, the impact of vitamin D supplementation on mitigating the risk of MI is debatable, as evidenced by interventional studies. Future research is thus warranted to showcase the promising approach of vitamin D supplementation in lowering the risk of MI, alongside investigating optimal dosing and evaluating long-term impacts on cardiovascular morbidity and mortality.

## Introduction and background

Vitamin D, also known as the sunshine vitamin, is currently one of the most debated risk factors in the development of myocardial infarction (MI). Although its role in the regulation of phospho-calcic metabolism is well established, its involvement in the evolution of cardiovascular diseases (CVDs) is yet to be fully understood. The active form, calcitriol (1,25 dihydroxycholecalciferol), is obtained via a series of hydroxylation reactions of 7-dehydrocholesterol, which in turn is synthesized from cholesterol present in the human epidermis, with the help of ultraviolet radiation. Calcitriol binds to the vitamin D receptor (VDR) located inside the cell nucleus, where it modulates the transcription of more than 200 genes [1-4]. VDR is a nuclear receptor, which is specifically activated by lipophilic molecules the size of cholesterol [5]. The lipophilic nature of vitamin D reduces the need for supplementary steps of signal transduction for gene regulation. Most human body tissues and different varieties of cells are sensitive to calcitriol due to the widespread presence of VDR [6]. The VDR, which are expressed in enterocytes, osteoblasts, parathyroid glands, and distal renal tubule cells, regulate phospho-calcic metabolism. Recent studies have manifested that VDRs also reside in the endothelial cells, lymphocytes, macrophages, smooth vascular muscle cells, beta-pancreatic cells, and cardiomyocytes, through which vitamin D3 generates its cardiovascular effects [7-9].

**Figure 1:**
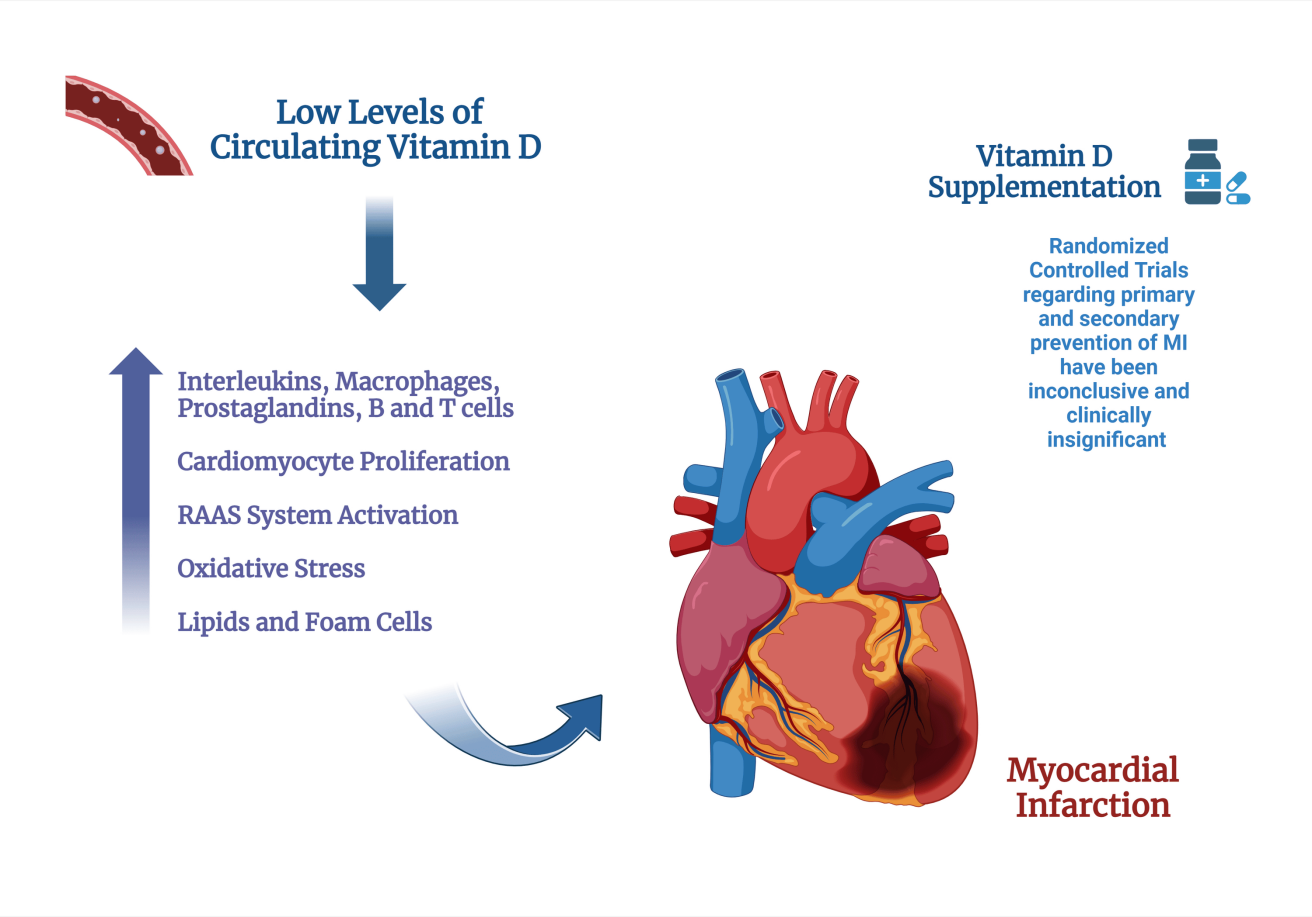
Vitamin D and myocardial infarction (MI) supplementation The figure has been made using biorender.com RAAS: Renin Angiotensin Aldosterone System

CVDs, including MI, are major contributors to the global burden of mortality and morbidity, despite the implementation of effective preventive and therapeutic strategies. In 2021, more than 30 million patients suffered ischemic heart disease, and approximately nine million died. The prevalence of MI among individuals aged above 60 is 9.5% [10,11]. Apart from conventionally recognized risk factors associated with the development of acute myocardial infarction (AMI), the recent emergence of potentially treatable risk factors has stimulated the interest of the medical faculties [12,13]. The Third National Health and Nutrition Examination Survey (NHANES III) depicted that vitamin D deficiency is frequently encountered all over the United States, and since it is easily treatable, it is of utmost importance to understand the association between vitamin D deficiency and MI [14,15]. In the majority of populations studied, there is a noticeable increase in the rate of cardiovascular mortality at higher latitudes. This trend typically intensifies in the colder winters, yet it decreases at higher altitudes. Previous studies have associated this pattern with the detrimental impact of hypovitaminosis D, a condition more prevalent in higher latitudes, cold winters, and lower altitudes [16-18]. The United States Endocrine Society guideline recommendations define normal vitamin D serum levels as being greater than or equal to 30 ng/mL. Vitamin D insufficiency is identified by levels ranging from 21 to 29 ng/mL, while deficiency is indicated by levels below or equal to 20 ng/mL. It is noteworthy that vitamin D deficiency is a prevalent nutritional inadequacy present globally among patients of all age groups, including children and adults [19-21]. About one billion people globally suffer from hypovitaminosis D, among which one-third of these patients have no recognizable risk factors (Figure 1) [22,23].

In this review, we provide a comprehensive overview of the available literature delineating the association between vitamin D deficiency and MI, proposed mechanisms of action, and therapeutic implications of vitamin D supplementation in diminishing the risk of CVD, to help researchers and clinicians elucidate diagnostic and therapeutic guidelines.

## Review

Proposed mechanisms

The proposed mechanism linking vitamin D to cardiovascular health involves various factors. Vitamin D executes a significant role in cellular growth, DNA repair, cell differentiation, apoptosis, and oxidative stress [24,25]. The VDR is present in a variety of tissues, such as cardiomyocytes and vascular smooth muscle. VDR, which is activated by vitamin D, affects gene expression, proliferation, and differentiation. Plaque formation and stability are postulated to be influenced by vitamin D levels, as increased intima thickness and coronary artery calcification have been shown to be linked to vitamin D deficiency [26-28]. Vitamin D also inhibits the development of foam cells from macrophages, thrombosis, uptake of cholesterol by the macrophages, facilitates high-density lipoprotein (HDL) transport, improves nitric oxide production, and aids vascular repair, all of which are shown to be protective against cardiovascular events such as MI [29,30]. Low levels of circulating vitamin D have been linked with several CVDs, including hypertension, coronary artery disease (CAD), ischemic heart disease, stroke, and heart failure [31,32].

A fundamental reason for vitamin D deficiency causing cardiovascular disorders, such as MI, is that it controls inflammation, atherosclerosis, and endothelial dysfunction. Low levels of vitamin D can cause increased inflammation and endothelial dysfunction. This can further lead to atherosclerosis, which is one of the main risk factors for MI [33,34]. Vitamin D plays a role in the regulation of the synthesis of inflammatory cytokines and the prevention of the growth of pro-inflammatory cells, which play a crucial role in inhibiting the mechanisms responsible for atherosclerosis. Suppression of prostaglandin and cyclooxygenase pathways, downregulation of the renin-angiotensin-aldosterone system (RAAS), amplification of anti-inflammatory cytokines, lowering of cytokine-induced manifestation of adhesion molecules, and matrix metalloproteinase 9 all lead to atherosclerosis [35,36]. Vitamin D promotes downregulation of the RAAS system through a cis-DNA element in the promoter region of the renin gene. This inverse relation between vitamin D levels and the RAAS system explains the hypertension associated with vitamin D deficiency, thereby enabling endothelial dysfunction, leading to plaque formation, ultimately ending up in MI [37,38].

Vitamin D is known to have cardioprotective effects by regulating mitochondrial metabolism and energy supply [39]. It can influence insulin sensitivity and elevate the risk of cardiovascular events, such as heart attacks [40-42]. In calcium homeostasis, vitamin D influences the opening of the L-type calcium channel and the activities of enzymes through non-genomic mechanisms, hence affecting smooth muscle contractility and tone, which contributes significantly to cardiovascular health [26]. Low vitamin D has been connected to heart problems, such as hypertrophy and remodeling of the left ventricle, both of which are risk factors for MI [43,44].

Concerning the female populations, vitamin D insufficiency or deficiency has been found to be linked with the development of cardiovascular disorders, especially MI, as a result of a complicated web of interactions between vitamin D and estrogen affecting the onset, duration, and prevention of heart attack in women. Estrogen has been revealed to have favorable effects on the cardiovascular system in female populations. Low levels of vitamin D can cause a drop in circulating estrogen, which is hypotensive and induces vascular relaxation. Due to low circulating estrogen, the risk of developing hypertension is increased, and protection against high-pressure-induced damage to arteries is reduced [45,46]. Furthermore, women's cardiovascular risks can be made worse following menopause by the reduction in estrogen levels, which, when combined with a vitamin D deficiency, makes them more vulnerable to MI [45,47]. Moreover, vitamin D is essential for protecting cardiomyocytes, especially in women. It has been exhibited that vitamin D and estrogen tend to protect cardiomyocytes by three main mechanisms, i.e., controlling mitochondrial activity, calcium homeostasis, and contractility. A person's susceptibility to cardiovascular disorders like MI, particularly in women, can be raised by cardiomyocyte hypertrophy, accelerated matrix turnover, and kinetic abnormalities when vitamin D levels are insufficient [47].

Observational studies

Association of Vitamin D Deficiency and MI

A case-control study in an Iraqi population of 222 patients (153 men and 69 women) between the ages of 22 to 80 years, with AMI and a control group of 225 sex- and age-matched individuals with no CAD, showed a significant level of vitamin D deficiency in AMI patients (95.9%), compared to the control group (78.4%). Moreover, male AMI patients had a higher rate of vitamin D deficiency than female AMI patients [48]. A similar case-control study from Bangladesh showed that low serum vitamin D is an independent risk factor for AMI. Risk was higher in serum vitamin D <20 ng/mL, compared to serum vitamin D >20 ng/mL. The enrolled study participants were categorized into three groups. Group A included patients with ST-segment elevation myocardial infarction (STEMI), Group B comprised non-ST-segment elevation myocardial infarction (NSTEMI), and Group C included age- and sex-matched individuals without AMI. The mean values of serum vitamin D were 20.17 ng/mL, 20.8 ng/mL, and 24.77 ng/mL, respectively, in STEMI, NSTEMI, and control groups. Vitamin D was considerably low in STEMI and NSTEMI groups compared to the control group (p < 0.001 and p = 0.004) [49].

Supporting the above studies, a case-control study from Saudi Arabia revealed that subjects with vitamin D deficiency (serum 25(OH)D <20 ng/mL) were 6.5 times more prone to experience coronary heart disease than the subjects with sufficient vitamin D levels (serum 25(OH)D ≥20 ng/mL; 95% CI: 2.7-15, p < 0.001). The limitations are that the measurements of circulating vitamin D levels were not conducted prior to diagnosing the cases [50]. A descriptive cross-sectional study from India showed that vitamin D deficiency is highly prevalent in the Indian subcontinent, and serum calcium levels are low in CAD patients compared to controls (p < 0.0001). Among the cases, 51.2% of the patients were vitamin D deficient, and 44.6% of patients had insufficient vitamin D levels. Serum vitamin D levels were categorized into deficient (<30 nmol/L), insufficient (30-75 nmol/L), and sufficient (>75 nmol/L) groups. Limitations include the descriptive approach of the study and the absence of randomization. The effect of unknown confounders on the observed association was not accounted for, and hence causality was not established. The external validity of the study was challenged due to the smaller sample size and data collection confined to a single center [51]. 

In a cross-sectional study conducted over a period of six months in Bangladesh by Chowdhury et al., 100 subjects from the Department of Cardiology were evaluated. The study found that 80% of cases, comprising patients with both STEMI and NSTEMI, had serum 25(OH)D levels of less than 20 ng/mL, indicating moderate to severe deficiency. Despite the small sample size and shorter follow-up period, the difference between the two groups was found to be statistically significant (p = 0.04) [52]. Similarly, in a study performed in 2015 by Roy et al., focusing on the association of vitamin D deficiency as a risk factor for AMI in Indians, a high prevalence of vitamin D deficiency was observed, with severe vitamin D deficiency (<10 ng/mL) being noted in 79.2% of cases and in 46.7% of controls (p < 0.001). Despite the findings, limitations of the study included poor representation of women and insufficient data on skin hyperpigmentation and the diet of the individuals. Interestingly, higher-than-expected vitamin D deficiency was observed in the controls as well [53]. 

In line with these, Karur et al. proved that 83.5% of patients with strong evidence of AMI exhibited abnormally low levels of 25(OH)D, notably with 67.5% having levels below 20 ng/mL. Nonetheless, a limitation faced by Karur et al. was the lack of consideration for seasonal variations, as the study was not conducted throughout the year [54]. A retrospective evaluation of STEMI patients by Uguz et al. revealed a negative correlation between vitamin D levels and thrombus load, with a p-value of 0.018 [55]. Agreeing with the data given above, in a meta-analysis of eight observational studies with 9,913 participants, of which 3,411 were MI patients, vitamin D levels were discovered to be significantly lower in the MI group compared to the control group, with a statistical significance of p = 0.007. A study depicted that sufficient vitamin D levels are cardioprotective in the American and Asian populations [56]. 

In Female Population

In the female population specifically, more compelling evidence of the role of vitamin D in the development of MI was discovered from the limited studies available. In 2022, a cross-sectional study among 100 post-menopausal women aged 45 to 70 years, consisting of 25 controls, 25 with MI, 25 with osteoporosis alone, and 25 with both osteoporosis and MI, was undertaken by Majeed et al. [57]. It was revealed that mean vitamin D levels were 5.30 ± 0.70 ng/mL in women with heart disease alone and decreased further to 4.10 ± 0.30 ng/mL in those with both heart disease and osteoporosis, compared to the control group with a mean of 9.20 ± 1.30 ng/mL. Hence, it was interpreted that vitamin D levels are significantly decreased (p < 0.05) in post-menopausal women with MI [57]. Likewise, a Chinese case-control study among 93 consecutive female patients aged 50 to 79 years old undergoing coronary angiography for evaluation of CAD and 119 age-matched controls showed that the risk of CAD was three-fold higher in those with 25(OH)D <10 ng/mL compared to those with 25(OH)D ≥20 ng/mL. Vitamin D-deficient individuals had a 52.8% CAD prevalence, whereas vitamin D-insufficient patients had a 31.8% prevalence (p < 0.001). A small sample size and no follow-up studies were the implied limitations [58].

Ma et al. demonstrated a significant decrease in carotid intima-media thickness (CIMT) and fewer carotid plaques in normotensive post-menopausal women with serum 25(OH)D in the fourth quartile, compared to those in the lower quartiles (p = 0.0039). This three-year study involving 671 women utilized radiological evaluation of carotid arteries, identifying thickening exceeding 50% of surrounding tissue or a focal thickness exceeding 1.5 mm protruding into the lumen. Hence, it was concluded that an inverse relationship between 25(OH)D and carotid atherosclerosis exists. Limitations of the study include reliance on a single morning fasting value of 25(OH)D, without considering albumin and vitamin D binding protein levels, as well as insufficient data on confounding factors such as physical activity, duration of sunlight exposure, and socio-economic status [59].

In a systematic review of multiple observational studies, a higher prevalence of metabolic syndrome was seen in women with vitamin D deficiency compared to those with normal vitamin D levels. This correlation included factors such as obesity, high blood pressure, elevated triglycerides, and HDL deficiency, all of which are significantly associated with the development of MI [60]. Similar findings were also observed in post-menopausal women with vitamin D deficiency, wherein there was a higher prevalence of metabolic syndrome, hypertriglyceridemia, and HDL deficiency, leading to a higher risk of cardiovascular mortality [61].

Vitamin D Intake

Intake of vitamin D seemed to yield favorable outcomes as well. Acharya et al. provided evidence based on a retrospective, observational case-control study, where a group of participants who had taken vitamin D supplements and maintained serum levels above 20 ng/mL exhibited a significantly lower risk of all-cause mortality. Moreover, pronounced results were observed in those with serum levels higher than 30 ng/mL, showing a reduced risk of MI compared to untreated patients with levels lower than 20 ng/mL. Limitations included the inability to determine the duration and compliance of treatment, and not accounting for racial differences [29].

Post-MI Mortality Risk

In a prospective cohort analysis conducted by Cruijsen et al., post-MI patients aged between 60 and 80 years were followed up for a period of 12 years, and their serum vitamin D levels were divided into tertiles, with cut-offs at 18.3 and 26.2 ng/mL. The results demonstrated that, with rising vitamin D levels across tertiles, there was a corresponding decreased risk of recurrent CVDs and mortality rates. Notably, the hazard ratios were 0.76 for the mid tertile and 0.67 for the upper tertile, compared to the lower tertile. This inverse relationship was further highlighted among those with higher calcium intake [62]. A consecutive cohort of 450 patients admitted for STEMI and treated with percutaneous coronary intervention (PCI), divided according to tertile values of 25(OH)D, shows that lower levels of vitamin D are independently associated with impaired reperfusion [63]. Indeed, patients with lower vitamin D levels exhibited an increased hazard ratio for follow-up all-cause mortality, as evidenced by Naesgaard et al., thereby highlighting it as a marker of morbidity, including the risk of sudden cardiac death in post-MI participants. However, the authors noted a limitation: power calculations cannot be directly applied to observational studies, and there is a high potential for confounding. Therefore, conclusions drawn are only tentative and not definitive [64].

Risk in STEMI vs. NSTEMI

Safaie et al. conducted a prospective case-control study to understand the association between 25(OH)D levels and the type of MI. A total of 88 patients were enrolled in the study, including 40 patients in the STEMI group and 48 patients in the NSTEMI group. Hypovitaminosis was found in 59.1% of the participants, with a prevalence rate significantly higher in STEMI patients (77.5% vs. 43.7%, p = 0.001). The blood plasma levels of 25(OH)D were notably lower in the STEMI group compared to the NSTEMI group: 13.5 ± 7.7 and 24.3 ± 14.9, respectively. Vitamin D deficiency proved to be a potentially major risk factor in the development of STEMI type (odds ratio = 8.1, 95% CI: 2.3-28.2, p = 0.001) [65].

Seasonal Differences

Tokarz et al. conducted a study with 59 patients diagnosed with an uncomplicated MI, recording their 25(OH)D levels throughout the year to see whether there would be changes in the January-March time frame compared to the September-December time frame. Out of the 59 patients enrolled in the study, 53 patients (89.9%) had vitamin D levels recorded below 20 ng/mL, and no patient had the recommended reference range. This study was conducted in Poland, which had altered values to indicate suboptimal vitamin D levels, whereas in the United States, levels below 10 ng/mL are considered abnormal. Seasonal changes were recorded, with the highest levels of vitamin D present during the October-December time frame and the lowest levels present during the first quartile of the year, from January to March. Even with the discrepancies between normal and abnormal vitamin D levels, this trend was also seen in the northern hemispheres, where sunlight is less intense. The study also reported that normoglycemic patients had significantly higher levels of vitamin D (9.2 (2.3-16.8) ng/mL) compared to patients with impaired glucose tolerance (2.3 (2.3-3.9) ng/mL) or diabetes mellitus (8.5 (2.5-13.3) ng/mL) (p = 0.01) [66]. All observational studies associating vitamin D deficiency with MI have been summarized in Table 1.

**Table 1:** Association between vitamin D levels and myocardial infarction: observational studies MI: Myocardial Infarction; AMI: Acute Myocardial Infarction; CAD: Coronary Artery Disease; MS: Metabolic Syndrome; HDL: High Density Lipo-protein; TGL: Triglycerides; BPs: Blood Pressures; STEMI: ST-Segment Elevated Myocardial Infarction; NSTEMI: Non-ST-Segment Elevated Myocardial Infarction

Author	Year	Type of study	Vitamin D deficiency cut-off	Summary of findings
Chowdhury et al. [52]	2023	Cross-sectional	<20 ng/mL	Mean levels of vitamin D were remarkably lower in patients with AMI as compared to patients without AMI.
Majeed et al. [57]	2023	Cross-sectional	-	Postmenopausal women with vitamin D deficiency were observed to be more prone to heart conditions and osteoporosis.
Cruijsen et al. [62]	2023	Cohort	<20 ng/mL	An inverse linear relationship was established between the risk of CVD mortality and serum vitamin D concentration. Risk decreased by 11% per 5 ng/dL elevation in vitamin D levels. Calcium supplementation and exercise augmented the effect.
Uguz et al. [55]	2022	Retrospective cohort	<20 ng/mL	Low vitamin D serum concentration is inversely associated with markedly pronounced thrombus burden and impeded blood flow in the coronary blood vessels among patients of STEMI.
Akter et al. [49]	2021	Case-control	<20 ng/mL	The risk of developing AMI was significantly elevated among participants with serum vitamin D levels of less than 20 ng/mL in comparison to serum vitamin D of more than 20 ng/mL.
Acharya et al. [29]	2021	Case-control	<20 ng/mL	A lower incidence of MI was noticed among participants with serum vitamin D concentration above 30 ng/dL. The risk of all-cause mortality was considerably reduced among participants with serum vitamin D levels above 20 ng/dL and 30 ng/dL.
Verdoia et al. [63]	2021	Consecutive cohort	<20 ng/mL	Decreased levels of serum vitamin D were established as an independent predictor of compromised reperfusion in patients of STEMI treated with PCI.
Amen and Baban [48]	2020	Case-control	<20 ng/mL	Vitamin D deficiency was found to be significantly positively correlated with the incidence of AMI.
Xu et al. [58]	2020	Case-control	<10 ng/mL	The frequency of CAD was observed to be significantly higher among patients with vitamin D deficiency and insufficiency.
Maroufi et al. [60]	2020	Systemic review	-	High frequency of metabolic syndrome and CVD risk factors including elevated BP, BMI, and TG and low HDL is linked with vitamin D deficiency in the female population.
Akhtar et al. [51]	2019	Descriptive cross-sectional	<30 nmol/L	Vitamin D deficiency was not found to be associated with CAD as average values of vitamin D levels were not statistically different. Serum calcium concentrations were lower among individuals with CAD.
Schmitt et al. [61]	2018	Cross-sectional	<20 ng/mL	Low serum vitamin D concentration is correlated with increased occurrence of CVD risk factors including metabolic syndrome and hypertriglyceridemia predominantly among postmenopausal women.
Safaie et al. [65]	2018	Prospective case-control	< 30 ng/mL	Vitamin D deficiency was found to be attributable to STEMI as the frequency of hypovitaminosis D was remarkably superior among patients of STEMI as compared to NSTEMI.
Huang et al. [56]	2017	Meta-analysis	<15 ng/mL	Higher prevalence of vitamin D deficiency was observed among patients with MI. It was postulated that higher levels of vitamin D may contribute as a protective factor against the development of MI.
Aljefree et al. [50]	2016	Case-control	<20 ng/mL	Subjects with vitamin D deficiency were 6.5 times more likely to suffer CHD than the subjects with adequate vitamin D status.
Tokarz et al. [66]	2016	Cohort studies	<20 ng/mL	Correlation between vitamin D deficiency and the occurrence of MI was established. Lowest levels of vitamin D were recorded during the first quarter of the year. Higher levels of vitamin D were observed among patients with normal blood glucose levels as compared to patients with diabetes mellitus.
Roy et al. [53]	2015	Case-control	<30 ng/mL	Markedly decreased levels of vitamin D were found among participants with acute MI, as compared to normal individuals. The frequency of comorbid conditions, including diabetes, hypertension, and hypercholesterolemia, as well as tobacco and alcohol intake, was higher among patients with AMI.
Naesgaard et al. [64]	2015	Cohort	<16 ng/mL	Risk of all-cause mortality is increased among patients of ACS with lower levels of serum vitamin D, specifically in women. Vitamin D deficiency is also associated with lower levels of omega-3 in the winter season.
Karur et al. [54]	2014	Cohort	<20 ng/mL	Higher prevalence of vitamin D among patients of AMI was associated with lower socioeconomic class, lack of exercise, smoking, elevated cholesterol, and diabetes.
Ma et al. [59]	2014	Cross-sectional	Quartiles: 12.4-30.7, 30.8 -40.5, 40.6-53.0, and 53.2-153.0 nmol/L	Post-menopausal women with normal blood pressure and glucose levels and higher serum 25(OH)D levels, particularly those in the fourth quartile, exhibited a lower prevalence of carotid plaque and decreased carotid intima-media thickness (CIMT).

Randomized controlled trials of vitamin D supplementation

A randomized, multicenter, double-blind, placebo-controlled trial of calcium and vitamin D supplementation for the prevention of colorectal adenomas was conducted in the United States, in which patients between the ages of 45 to 75 years were recruited, who had undergone removal of at least one colorectal adenoma within 120 days prior to enrollment, with no residual disease. These patients were divided into four groups, where each patient had to take two tablets, which could either be: 1000 IU of vitamin D3, 1200 mg of calcium, both agents, or placebo. The rate of adenoma occurrence was studied, along with adverse effects such as MI, with or without revascularization. Out of 1130 patients who were categorized into the vitamin D group, eight patients developed MI, whereas seven patients who were not taking vitamin D had MI as an adverse effect. Hence, supplementation of cholecalciferol in patients with adenomas did not yield any significant difference in the occurrence of MI [67].

A randomized controlled trial from 2008 to 2015, led by Jorde et al. at the University Hospital of North Norway, studied 511 individuals with prediabetes, with six-monthly checkups. They received either vitamin D supplementation (20,000 IU/week) or a placebo. After the intervention, the vitamin D group's serum levels rose significantly to 122 nmol/L, while the placebo group remained stable. During the trial, the adverse outcome of coronary infarction was also observed, showing that three individuals among the 256 (1.17%) in the vitamin D group and six among the 255 (2.35%) in the placebo group suffered MI [68].

In 2017, Scragg et al. conducted a vitamin D assessment study in which 5108 participants, aged between 50 and 84 years, were randomized to receive vitamin D3 (n = 2558) or placebo (n = 2550). The intervention involved an initial high dose of 200,000 IU of vitamin D3, followed by a monthly dose of 100,000 IU, or placebo, for around 3.3 years. MI was studied as a secondary outcome in this randomized, double-blinded, placebo-controlled trial. The results showed that, in the vitamin D group, 34 cases versus 31 cases in the placebo group were recorded. Therefore, it was concluded that a monthly high dose of vitamin D intake does not prevent MI [69].

A placebo-controlled randomized trial was conducted in 2011, ending in 2017, by Manson et al., with 25,871 participants across the United States, with a mean age of 67.1 years and no prior history of cancer or CVD, to observe the effect of 2000 IU of vitamin D and 1 g of omega-3 fatty acids per day on the prevention of cancer and CVD. An observation of 345 participants having an outcome of MI was made, as they underwent follow-up at six months and then annually, among whom 169 belonged to the vitamin D group and 176 to the placebo group, leading to a hazard ratio of 0.96 (0.78-1.19). Furthermore, for a total of 39 patients, MI led to death, among whom 24 were from the vitamin D group, while 15 had been provided with a placebo, showcasing a hazard ratio of 1.60 (0.84-3.06). It was concluded that vitamin D supplementation did not affect lowering the incidence of MI compared to those given a placebo [70].

Hypovitaminosis D escalates the risks of mortality in individuals with chronic kidney disease. The J-DAVID Randomized Clinical Trial in Japan tried to determine the effect of VDR activators in reducing the risk of cardiovascular events in patients receiving maintenance hemodialysis. The treatment included 0.5 micrograms of oral alfacalcidol per day in the vitamin D group versus treatment without VDR activators in the control group. AMI incidence was found in 10 people in the interventional group (n = 488) versus 11 in the control group (n = 476), which was found to be statistically insignificant [71].

To study the effectiveness of calcifediol in lowering mortality in patients with vitamin D insufficiency on hemodialysis compared to standard therapy, a randomized, open-label trial in a 1:1 ratio was conducted by Morrone et al. between 2012 and 2014 in dialysis centers around Italy, which included 284 adults with vitamin D insufficiency (25(OH)D <30 ng/mL, vitamin D deficiency levels <15 ng/mL) undergoing hemodialysis. Among these patients, 143 received oral calcifediol (40 mcg) post-dialysis session thrice a week, and 141 received standard care. A median follow-up of 24 months (IQR, 12-30 months) was observed, where one participant in the calcifediol group and five participants in the control group experienced the primary outcome of nonfatal MI, having a hazard ratio of 0.20 (0.02-1.67), while two participants from each group encountered fatal MI, leading to a hazard ratio of 0.94 (0.13-6.65). It was therefore concluded that calcifediol supplementation had indeterminate effects on MI outcomes [72].

A five-year randomized trial, led by Virtanen et al., investigated the effects of vitamin D3 supplementation on CVD and cancer incidences. The study included 2495 male participants aged ≥60 years and postmenopausal women aged ≥65 years from a general Finnish population, without a previous history of CVD or cancer. The study divided the population into three groups in a 1:1:1 ratio, which included those receiving either placebo, 1600 IU/day, or 3200 IU/day of vitamin D3. During the mean follow-up of 4.3 years, 56 participants were diagnosed with an MI, among whom 18 participants were from the placebo and the 1600 IU/day groups each, while 20 were from those receiving 3200 IU/day, leading to a combined hazard ratio of 0.98 (0.56-1.71). While the nominal p-value for interaction was statistically significant in the analyses by gender, the observed difference in hazard ratios was evident only in the 1600 IU/day vitamin D group, with women experiencing a higher event rate. However, there were no appreciable differences in the event rates of MI between the placebo arm and the two vitamin D arms [73].

A study was performed to assess the impact of micronutrients on cardiac health. This study is composed of a systematic review and meta-analysis of RCTs. A total of 883,627 participants were assessed with 27 different types of micronutrients, including but not limited to vitamin D, selenium, folic acid, magnesium, zinc, and melatonin. The study concluded that some micronutrients, such as melatonin, have an intermediate effect on cardiac health, whereas others, such as vitamin D, along with vitamin C and vitamin E, showed no risks in developing cardiac events, particularly MI [74].

From 2012 to 2022, a double-blind, placebo-controlled randomized trial led by Joseph et al. across 86 centers in nine countries involved 5670 participants without vascular disease but at increased cardiovascular risk. Participants were randomized 1:1 to receive either vitamin D (60,000 IU monthly) or placebo. The trial followed up with visits at six weeks, three, six, nine, and 12 months, then every six months until its conclusion. The outcomes studied included infarction, stroke, cancer, fracture, or fall. Among participants receiving vitamin D, 21 experienced an MI, compared to 19 in the placebo group, resulting in an MI hazard ratio of 1.11 (0.60-2.07) for vitamin D versus placebo [75].

In the D-Health trial, the effect of vitamin D supplementation in older Australian adults, with monthly dosing of 60,000 IU versus placebo, was studied over a period of five years. Thompson et al. found that the hazard ratio for MI was 0.81, with a 95% CI of 0.67-0.98; hence, the rate of MI was lower in the vitamin D group. These findings were more evident in people taking cardiovascular drugs for either prevention or treatment [76]. All randomized controlled trials have been summarized below in Table 2.

**Table 2:** Association between vitamin D supplementation and myocardial Infarction: randomized controlled trials MI: Myocardial Infarction; CKD: Chronic Kidney Disease; CVD: Cardiovascular Disease; IU: International Units

Author name	Year of publication	Amount of vitamin D supplemented	Control	Risk ratio	Conclusion
Baron et al. [67]	2015	Vitamin D3 (1000 IU) daily plus calcium (1200 mg) daily	Calcium (1200 mg) daily	1.14	Supplementation of cholecalciferol in patients with colorectal adenomas did not yield any significant difference in the occurrence of MI.
Jorde et al. [68].	2016	Vitamin D3 (20,000 IU) weekly	Placebo	0.50	Vitamin D supplementation in pre-diabetic patients does not lower the risk of development of coronary infarction.
Scragg et al. [69]	2017	Vitamin D3 initial (200,000 IU) then vitamin D3 (100,000 IU) monthly	Placebo	0.90	The risk of development of myocardial infarction is not mitigated by a high dose of vitamin D intake every month.
Manson et al. [70]	2018	Vitamin D3 (2000 IU) daily	Placebo	0.96	General outcomes did not show any effects of vitamin D3 supplementation on reducing the risk of heart diseases, especially MI, and deaths due to malignancies.
Shoji et al. [71]	2018	Oral alfacalcidol 0.5 μg daily	Placebo	0.89	Use of oral alfacalcidol does not reduce the risk of myocardial infarction in CKD patients without secondary hyperparathyroidism.
Morrone et al. [72]	2022	Vitamin D3 40 mcg after each dialysis	Standard care	0.39	Calcifediol supplementation had indeterminate effects on cardiovascular outcomes among patients undergoing hemodialysis.
Virtanen et al. [73]	2022	Vitamin D3 1600 IU daily or 3200 IU daily in 1:1	Placebo	1.05	Vitamin D3 supplementation had no effect on lowering the prevalence of CVD and cancer among elderly patients.
Joseph et al. [75]	2023	Vitamin D3 60000 IU monthly	Placebo	1.11	High-dose vitamin D supplementation was not proven effective in diminishing the risk of cardiovascular events including MI, and fractures.
Thompson et al. [76]	2023	Vitamin D3 60000 IU monthly	Placebo	0.81	The overall incidence of cardiovascular adverse outcomes was lower among patients taking vitamin D; however, the findings were rendered statistically insignificant.

Several meta-analyses performed on randomized controlled trials reflected similar outcomes. Vitamin D supplementation was not statistically associated with a lower risk of cardiovascular mortality and morbidity, including MI and stroke, and was rendered clinically insignificant for the prevention or treatment of CVDs. However, some studies showed a slight decrease in all-cause mortality, and certain limitations of individual studies were not accounted for in the analyses. Few studies showed promising results but lacked the statistical guardrail that aided clinicians in differentiating chance from causality [77-85].

Discussion

Extensive observational studies have indicated that a deficiency in vitamin D may heighten the likelihood of CVD, and a potential association exists between hypovitaminosis D and the occurrence of MI. Further research is warranted to scrutinize factors that could potentially alter the effect or serve as confounding variables. These factors include other comorbid conditions in patients, medication use, level of skin pigmentation, risk factors including blood cholesterol levels, triglyceride levels, smoking habits, hormonal changes between males and females, socioeconomic status, and sedentary lifestyle. Overall, many studies included a small population of people per group, which undermined the power of the study. The time period during which the studies were conducted had potentially altered the results, as winter and summer months lead to varied sun exposure, producing varying amounts of vitamin D throughout the year. Another bias was the limited control group to compare individuals with normal vitamin D levels to those with abnormal levels in MI. One study noticed that patients were previously diagnosed with other comorbidities, including diabetes mellitus, which has also previously been linked with vitamin D deficiency. 1a-hydroxylase conversion of inactive vitamin D to active vitamin D is impaired and is associated with impaired fasting glucose. Other findings revealed that patients of Indian ethnicity and darker-skinned individuals seemed to have a higher prevalence of vitamin D deficiency. Low vitamin D levels were also seen in lower socioeconomic status patients, possibly due to a lesser degree of sun exposure and poor intake of nutritional supplements to boost vitamin D uptake. Women, especially postmenopausal women, are recommended to take supplemental vitamin D to prevent future problems of osteoporosis and bone fractures, which could be a competing factor for why a large percentage of the study group was vitamin D deficient. Patients with impaired glucose tolerance and diabetes mellitus were also reported to have significantly lower vitamin D levels compared to those with normoglycemia. There is a scarcity of research to understand whether vitamin D deficiency impairs glucose tolerance or whether supplemental vitamin D could improve glycemic control [28-46].

In the past, a health professionals' follow-up study followed 18,225 men, showing that vitamin D-deficient men had an increased risk of MI compared to those with sufficient vitamin D levels (relative ratio: 2.42; 95% CI: 1.53-3.84; p < 0.001) [86]. A cohort study performed in rural Sweden yielded similar results, indicating that participants in the highest quartile of serum vitamin D levels had halved the relative risk of ischemic heart disease compared to participants in the lowest quartile. However, no dose-response relationship was established in the four quartiles. Potential confounders, including smoking status, blood pressure, triglyceride levels, and physical work, were accounted for but showed statistically insignificant results [87]. However, other studies, such as the MINI-Finland Health Survey, were able to stratify season and traditional CAD risk factors, which then eliminated any association between vitamin D and coronary heart disease (p = 0.20) [45]. A meta-analysis was able to depict a linear reverse dose-response relationship between circulating levels of 25(OH)D ranging from 20 to 60 nmol/L and the risk of CVD [88].

The anti-atherosclerotic characteristics of vitamin D are reported as activating nitric oxide synthase in endothelial cells, which acts as a vasodilator. It also functions as an anti-inflammatory factor by inhibiting interleukins (IL-6 and IL-8). Cyclooxygenase-2 (COX-2) expression is decreased, and 15-hydroxyprostaglandin dehydrogenase (15-PGDH) production is stimulated, inhibiting intercellular adhesion molecule (ICAM-1), vascular cell adhesion molecule (VCAM-1), and platelet endothelial cell adhesion molecule (PECAM-1) through nuclear factor kappa-light-chain-enhancer of activated B cells (NF-kB), thereby diminishing thrombosis formation. Insulin resistance, B-cell dysfunction, dyslipidemia, and the RAAS system are some of the other indirect processes that affect anti-atherosclerotic properties [89].

The randomized controlled trials conducted to evaluate the efficacy of vitamin D supplementation to minimize the risk of MI showed disputable results in terms of primary prevention. Some studies depicted a relative risk reduction in MI due to vitamin D supplementation; however, the results were not statistically significant, and the confidence intervals were consistent with the null findings. Some studies also revealed that participants who took statins or other cardiovascular drugs while taking the vitamin D supplement seemed to have a reduced risk of major cardiovascular events. The study did not find any interactions with age, sex, or BMI. Although the number of stroke-like events was lower, there is no evidence to advocate that vitamin D has a protective effect against cardiovascular events. The possible advantage of vitamin D administration during the initial phase of AMI has not been completely explored and is one area that needs to be studied [47-56]. Nevertheless, one recent study published that one-year supplementation of vitamin D, concurrently with optimal therapy, led to significant improvement in left ventricular function among patients with heart failure due to left ventricular systolic dysfunction. Interestingly, ischemic heart disease was the fundamental cause among 60% of these patients, promising potential advantage among post-AMI patients with left ventricular dysfunction [90-94].

Future directions

The majority of the studies performed in the past were observational studies, indicating a theoretical association between blood vitamin D levels and MI. However, it is unclear whether low vitamin D levels are a risk factor or a marker of AMI and its application in a clinical setting. Hence, it is imperative that future studies focus on vitamin D’s protective ability and explore whether there is a clinically significant association. Moving forward, the following directions could enhance our understanding and potentially pave the way for more effective interventions. Exploring the mechanisms by which vitamin D affects cardiovascular health is crucial. In-depth studies are required to unravel the precise molecular pathways behind the anti-inflammatory, anti-thrombotic, and vascular protective effects of vitamin D. This will help in developing targeted therapies and interventions. The optimal dosage and serum levels of vitamin D for cardiovascular health are still subject to debate. Therefore, long-term studies with diverse populations should be conducted to establish clear dose-response relationships and identify specific thresholds for cardiovascular protection. Large-scale, randomized clinical trials are essential to determine the efficacy of vitamin D supplementation in reducing the risk of CVDs. These trials should include diverse populations, including individuals with varying baseline vitamin D status, to assess the potential differential effects and ascertain generalizability. Investigating the potential interactions between vitamin D and other micronutrients, such as calcium, magnesium, and omega-3 fatty acids, could provide valuable insights into synergistic or antagonistic effects on cardiovascular outcomes. The interplay between the effects of cardiovascular drugs and circulating vitamin D levels needs to be investigated. Understanding these interactions is crucial for optimizing dietary recommendations and supplementation strategies. Personalized medicine approaches, including genetic profiling and consideration of individual lifestyle factors, can refine our ability to predict an individual's responsiveness to vitamin D supplementation in terms of cardiovascular risk reduction. Tailoring interventions based on these factors may enhance efficacy and minimize adverse effects. Population-based interventions aimed at improving vitamin D status through strategies such as fortification of foods, public health campaigns promoting sunlight exposure, and supplementation programs in high-risk groups could have significant implications for reducing the burden of CVDs on a global scale. It is crucial to evaluate the effectiveness and cost-effectiveness of such interventions. Long-term studies with extended follow-up periods are vital to assess the long-term impact of vitamin D supplementation on cardiovascular morbidity and mortality. Understanding the sustained effects and potential risks associated with prolonged supplementation is essential for informing clinical guidelines. In summary, while the current research on vitamin D and CVD risk is substantial, many avenues for further exploration and refinement exist. By addressing these future directions, we can advance our knowledge, optimize interventions, and ultimately mitigate the global burden of CVD [1,95-102]. Vitamin D deficiency is particularly a cause of concern among low- and middle-income countries due to concerning food availability. Similarly, low- and middle-income countries also share a larger proportion of the burden of CVDs, including MI and associated mortalities. Hence, clinical trials should be performed to compare outcomes between high-, middle-, and low-income countries, and guidelines should be established appropriately according to the requirements of the region [103,104].

## Conclusions

Vitamin D deficiency is a newly emerging risk factor for CVDs, particularly for MI. Several observational studies have depicted a correlation between vitamin D deficiency prevalence and the occurrence of MI among patients; however, it is ambiguous whether it is a risk factor or a marker for the disease. Randomized controlled trials supplementing vitamin D to curtail the risk of MI have been deemed controversial and clinically debatable.

It is imperative to conduct longer longitudinal studies with large sample sizes across various ethnic groups to reveal appropriate data regarding the role of vitamin D in primary and secondary prevention of MI. The correlation between circulating levels of 25(OH)D and other risk factors, including blood cholesterol and glucose levels, needs to be studied. The current guidelines for vitamin D deficiency and insufficiency cut-offs are related to bone metabolism, and it is essential to establish accurate guidelines related to CVD for appropriate diagnostic and therapeutic implications in a clinical setting.
